# Supplementary material for: Evidence supported by Mendelian randomization: impact on inflammatory factors in knee osteoarthritis
Source: Front Med (Lausanne). 2024 May 28;11:1382836. doi: 10.3389/fmed.2024.1382836 (PMC11165061; doi:10.3389/fmed.2024.1382836)
Supplement: Supplementary file 1 [file Data_Sheet_1.ZIP › data/GCST90274810_5e6/810.forest.pdf]

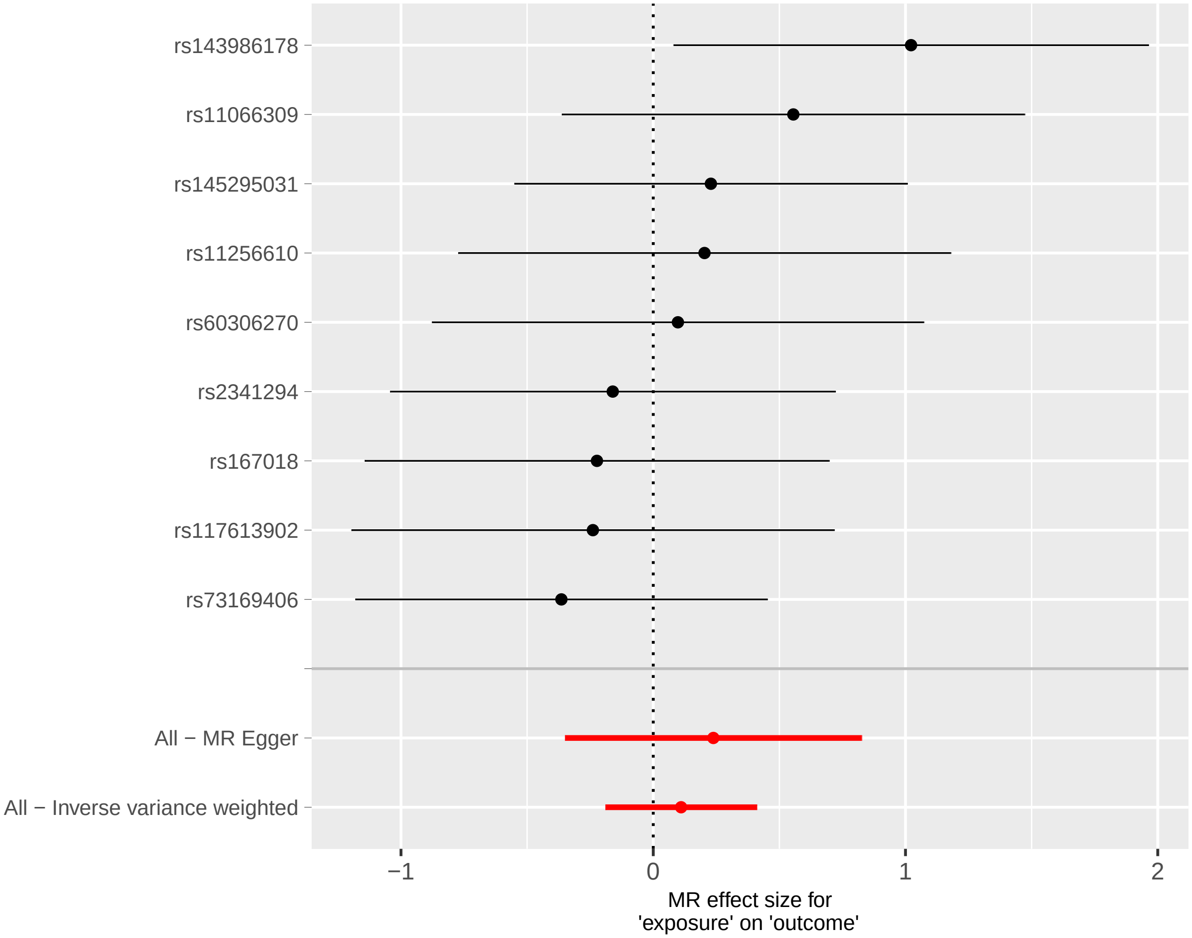
rs143986178
rs11066309
rs145295031
rs11256610
rs60306270
rs2341294
rs167018
rs117613902
rs73169406
All − MR Egger
All − Inverse variance weighted
−1
0
1
2
MR effect size for
'exposure' on 'outcome'
